# Faecal excretion of thorium by NORM workers

**DOI:** 10.1093/rpd/ncaf081

**Published:** 2025-07-15

**Authors:** Gregory S Hewson, Martin Ralph, Marcus Cattani

**Affiliations:** School of Medical and Health Sciences, Edith Cowan University, 270 Joondalup Drive, Joondalup, WA 6027, Australia; School of Medical and Health Sciences, Edith Cowan University, 270 Joondalup Drive, Joondalup, WA 6027, Australia; School of Medical and Health Sciences, Edith Cowan University, 270 Joondalup Drive, Joondalup, WA 6027, Australia

## Abstract

Exposure to thorium-bearing dust in industries handling and processing monazite and other minerals can pose radiological risks to workers. This study aimed to reassess historical faecal bioassay data collected over 10 d from two monazite plant workers using updated biokinetic and dosimetric models. Another objective was to evaluate the feasibility of faecal thorium bioassay for contemporary operations involving naturally occurring radioactive materials (NORM). The retrospective analysis found that the bioassay-derived thorium intakes were significantly higher than those estimated via personal air sampling. The effective dose estimates for the two workers were similar and ranged from 0.95 to 2.40 mSv over the 5-d exposure period, depending on the worker’s assumed mode of breathing. The study confirmed that faecal thorium bioassay remains a viable tool for monitoring workers exposed to insoluble thorium dust, but the timing of sample collection, individual physiology, and background dietary intake of NORM must be considered.

## Introduction

Operations involving the mining, separation, and processing of ores containing naturally occurring radioactive materials (NORM) may result in radiation doses to workers from the inhalation of dust. The committed effective dose (hereafter referred to as dose) to a worker depends on the duration of exposure, the activity concentration of the airborne radionuclides, the degree of equilibrium in the applicable naturally occurring decay chains, and factors related to the physical and chemical characteristics of the inhaled dust and individual worker characteristics. The assessment of dose by industry is typically conducted using personal air sampling (PAS) and applying approved or recommended dosimetric and biokinetic models to convert the inhalation intake estimate to dose [1]. Individual monitoring may also be undertaken, such as *in vivo* lung counting or *in vitro* analysis of excreta (e.g. urine, breath, or faeces), depending on the expected exposure levels and sensitivity of the bioassay methods. Hewson *et al.* [2] reported previous bioassay research in the Western Australian mineral sands industry and referred to similar work done in other countries. Much of the published bioassay work in NORM industries was conducted between the mid-1980s and 2000 [2].

With the publication of the International Commission on Radiological Protection (ICRP) Occupational Intake of Radionuclides (OIR) series [3–6], there is an opportunity to revisit past bioassay studies and reevaluate the data using the most recent biokinetic models, and hence review implications for intake and dose records. Faecal bioassay is an individual monitoring technique recommended following intake of insoluble thorium-bearing dust in industries associated with rare earth and mineral sands processing [7]. ICRP Publication 137 [4] shows that faecal bioassay for ^232^Th intake (via inhalation) is useful because the amount of ^232^Th excreted reaches a steady value within days and then drops substantially after the cessation of exposure. However, the ICRP also cautions about the need to consider ingestion of thorium in the diet, as this may complicate the interpretation of faecal bioassay results. Stradling *et al.* [8] concluded that, provided dietary excretion rates are low (<4 mBq d^−1^) and with a monitoring interval of 90 d, doses of <1 mSv y^−1^ after repeated intake can be assessed from thorium dioxide using a faecal bioassay. This conclusion was based on the application of the ICRP Publication 66 [9] model and default assumptions for type S (low-solubility) thorium dioxide.

This study aimed to reevaluate historical faecal thorium bioassay data using the newest biokinetic models and dosimetry models [3, 4], compare bioassay-derived and PAS-derived daily thorium intakes, and provide refined intake and dose assessments for two Western Australian monazite plant workers. The updated analysis of historical data was also used to assess the feasibility of further faecal thorium measurements on the contemporary mineral sands industry workforce based on their reported exposure records, and to assess implications for PAS monitoring strategies. The results of this study may also have implications for other operations processing NORM (e.g. rare earth, tantalum, or zircon processing) and where there is the potential for inhalation of insoluble thorium dust.

## Methods

### Faecal bioassay data

The bioassay data were extracted from a previously reported study by Terry *et al.* [10], which detailed the methods used for workers’ intake assessment by PAS techniques, faecal sample collection protocol, sample processing, and interpretation of the bioassay data using ICRP 30 dosimetric and biokinetic models [11]. Terry *et al.* [10] collected daily (24-h) faecal voiding from two long-term (>10 y) monazite plant workers over 10 d. Sample collection commenced on the first day back in the workplace (Day 1) after a 7-d isolation period from thorium dust exposure. After 5 d of occupational exposure, the workers had 2 d away from the workplace (Days 6 and 7) and then 3 d in a nonradioactive dust workplace (Days 8–10). The daily faecal samples were collected in plastic bags and frozen before delivery to the analytical laboratory. The samples were subsequently ashed and 1 g of the ash was dissolved in acids, diluted 200 times with 5% HCl, and quantified using inductively coupled plasma mass spectroscopy (ICP-MS). The limit of detection for thorium was quoted as 2 μg g^−1^ of faecal ash and further details of the ICP-MS operating conditions and sample preparation are described elsewhere [12].

The workers were ostensibly performing the same operational tasks over their five daily work shifts and the workplace environment was characterized by a mean airborne long-lived alpha activity of 0.55 ± 0.13 Bq_α_ m^−3^ (*n* = 5) from daily PAS measurements, and an activity median aerodynamic diameter (AMAD) of 14 μm, with a geometric standard deviation (GSD) of 3, from daily personal cascade impactor measurements [10].

Table 1 reproduces the daily measured thorium and uranium activities in faeces over the 10 monitoring days for both workers with some amendments. These data differ from those reported by Terry *et al.* [10] in that the thorium level is expressed as activity (in millibecquerel), and not weight, and the measured faecal uranium activities have also been included, as have the faecal thorium-to-uranium ratios. The uranium results were not included in the original study but have been published elsewhere [12]. A review of the original data set resulted in the correction of the data reported for worker E1. This worker provided daily samples on Days 1, 2, 4, 6, and 8 (incorrectly shown as days 2, 4, 6, 8, and 10 in the study by Terry *et al.* [10]).

**Table 1:** Measured thorium activity in faeces following a 5-d exposure to thorium-bearing (monazite) dust.

Monitoring day^a^	Worker E1^b^: activity excreted in faeces^a^				Worker E2^b^: activity excreted in faeces		
	Thorium^c^ (mBq)	Uranium^c^ (mBq)	Ratio Th:U		Thorium^c^ (mBq)	Uranium^c^ (mBq)	Ratio Th:U
1	120	37	3.2:1		100	37	2.7:1
2	610	87	7.0:1		610	100	6.1:1
3	–	–	–		900	110	8.1:1
4	1550	210	7.4:1		1710	250	6.9:1
5	–	–	–		1590	210	7.5:1
6	1140	160	7.1:1		2650	320	8.2:1
7	–	–	–		180	37	4.9:1
8	530	87	6.1:1		100	50	2:1
9	–	–	–		120	37	3.2:1
10	–	–	–		100	37	2.7:1
Total	3950	581	–		8060	1188	–

^a^Occupational exposure commenced on Day 1 and ceased at the end of Day 5. Worker E1 did not provide samples on days 3, 5, 7, 9 and 10.

^b^Workers E1 and E2 had a total faecal ash weight over the 10-d monitoring period of 78.1 and 72.6 g, respectively. The sample collected from worker E2 on Day 7 had a very low faecal ash weight of 4.1 g.

^c^Original data (in micrograms) converted to activity using ^232^Th specific activity of 4.07 × 10^−3^ Bq μg^−1^ and ^238^U specific activity of 12.4 × 10^−3^ Bq μg^−1^.

### Re-analysis of faecal bioassay data

Estimated intakes and corresponding committed effective doses were calculated using the Taurus internal dosimetry software (version 2.2) [13], which is based on the ICRP OIR series of publications [3–6]. The ‘retrospective calculation (data fitting)’ function of Taurus was used assuming, initially, a chronic intake of 10 μm AMAD (GSD = 3.0), type S (low-solubility) ^232^Th, heavy activity level, and the faecal excretion data in Table 1. An AMAD of 10 μm was conservatively applied to recognize the uncertainty in the particle size measurements by the personal cascade impactor. The data fitting of the ^238^U data for worker E2 was also investigated given the additional data available for that worker. The faecal excretion data for Days 7 and 8 for worker E2 were combined in the fitting analysis due to the low faecal ash weight for Day 7 (4.1 g), which implied that the sample was not representative of a 24-h collection. The absorption and respiratory tract parameters were initially selected as the ICRP OIR series defaults for ^232^Th and ^238^U unless otherwise noted. The bioassay fitting function of Taurus requires the selection of either a normal or a lognormal distribution of errors and measurement uncertainty values. A lognormal distribution was selected for this study, with a scattering factor, SF, (or GSD) of 2 based on sample collections over 10 d. The European Radiation Dosimetry (EURADOS) guidance [14] suggests SFs of 3 (range 2–4) and 2 (range 1.5–2.2) for faecal 24- and 72-h samples, respectively.

The ‘prospective bioassay’ function of the Taurus software was used to examine additional aerosol parameters and intake patterns, including work rosters and sample collection timing. Microsoft Excel was used to download and analyse data from Taurus and to produce daily faecal activity curves as a function of intake.

The original study [10] did not collect faecal samples from local, unexposed individuals; hence, the impact of thorium intake via diet and environmental exposure could not be addressed.

### Thorium intake derived from PAS

Based on the PAS-measured mean airborne long-lived alpha activity over the 5-d exposure period, the daily intake of ^232^Th for each worker was estimated as follows:

(0.55 Bq_α_ m^−3^/6) × 1.7 m^3^ h^−1^ × 8 h d^−1^ = 1.25 Bq d^−1^

where

6 = number of alpha emitters associated with decay of ^232^Th in secular equilibrium.

1.7 = breathing rate of worker.

8 = exposure hours per day.

The breathing rate was selected based on the workers being engaged in heavy work activities [15], such as physical brushing down of monazite separation equipment (e.g. air tables), manual housekeeping tasks, and accessing stairwells to various operating levels of the plant.

Note that the alpha activity contributed by the uranium decay chain was considered insignificant (<10%) in this analysis as monazite dust in Western Australian mineral sand plants will typically contains 6% thorium and 0.2% uranium [2].

## Results

### PAS-predicted faecal excretion rate

**Figure 1:**
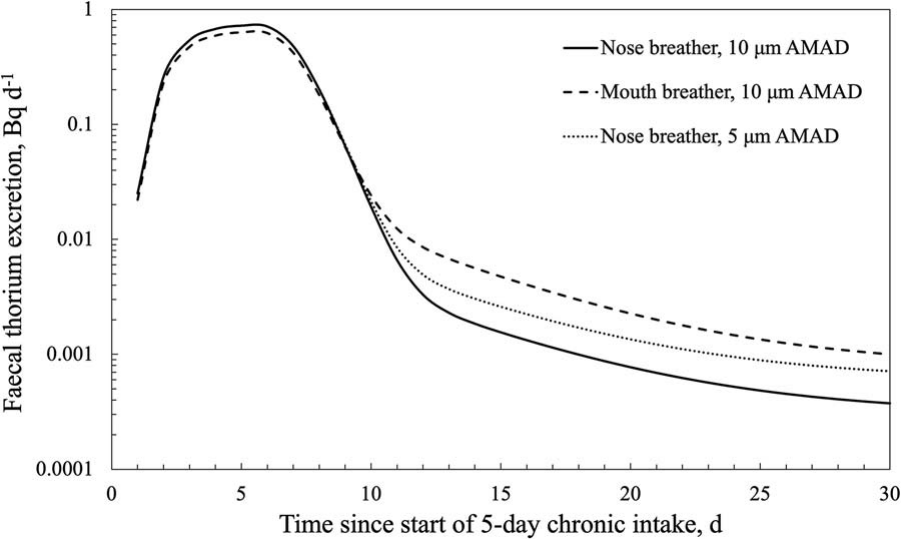
Predicted daily faecal thorium excretion following a 5-d intake of 10 and 5 μm AMAD, type S ^232^Th at 1.25 Bq d^−1^.

Using the Taurus software and applying a ^232^Th intake of 1.25 Bq d^−1^ for a 5-d exposure period and a 10-d bioassay monitoring period provided prospective bioassay excretion curves, as shown in Fig. 1. Three inhalation scenarios are shown; two scenarios relate to the inhalation of a 10 μm AMAD aerosol for two different modes of breathing (i.e. nose vs mouth). One scenario relates to the inhalation of a 5 μm AMAD aerosol via nose breathing. The curves were also extended to 30 d to show the rate of reduction in faecal thorium excretion after cessation of intake.

For the 10 μm AMAD intake scenarios, doses of 0.37 and 0.87 mSv over the 5-d exposure period were obtained for a nose-breathing and mouth-breathing worker, respectively. For the 5 μm AMAD, nose breather intake scenario, a dose of 0.67 mSv was obtained. The doses correspond to the thorium series in secular equilibrium in the inhaled mineral dust matrix.

### Predicted faecal thorium excretion over a work cycle

**Figure 2:**
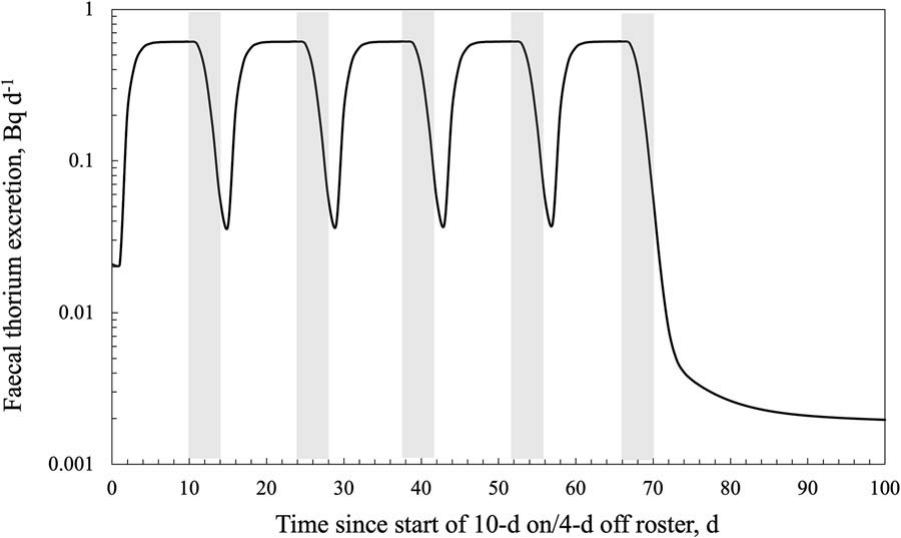
Predicted faecal thorium excretion over a work–rest cycle following 1 Bq d^−1^ intake of 10 μm AMAD, type S ^232^Th. Shaded areas show days away from work. Exposure ceased after the fifth roster cycle.

For protracted chronic intakes, the ICRP biokinetic models assume an average daily intake over the exposure period, without accounting for the lack of exposure to radionuclides during the days away from work over the employment period. The effect of the work cycle on bioassay predictions can be determined indirectly using the Taurus software by mathematically repeating a work–rest intake function over many cycles and summing the results for each day over the protracted exposure period. Figure 2 shows the results for faecal excretion of thorium following inhalation of a 10 μm AMAD, type S aerosol at 1 Bq d^−1^ for one of the work rosters typically used in the Australian minerals industry (i.e. 10 d on and 4 d off).

### Retrospective (data fitting) bioassay assessment

**Figure 3:**
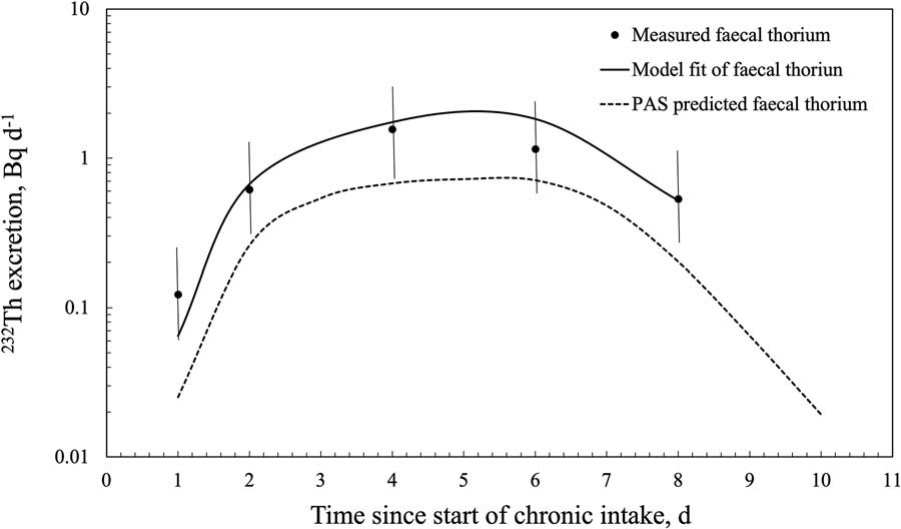
Retrospective fitting of faecal thorium bioassay data from worker E1 vs PAS-predicted faecal excretion. Uncertainty shown as a scattering factor of 2.

**Figure 4:**
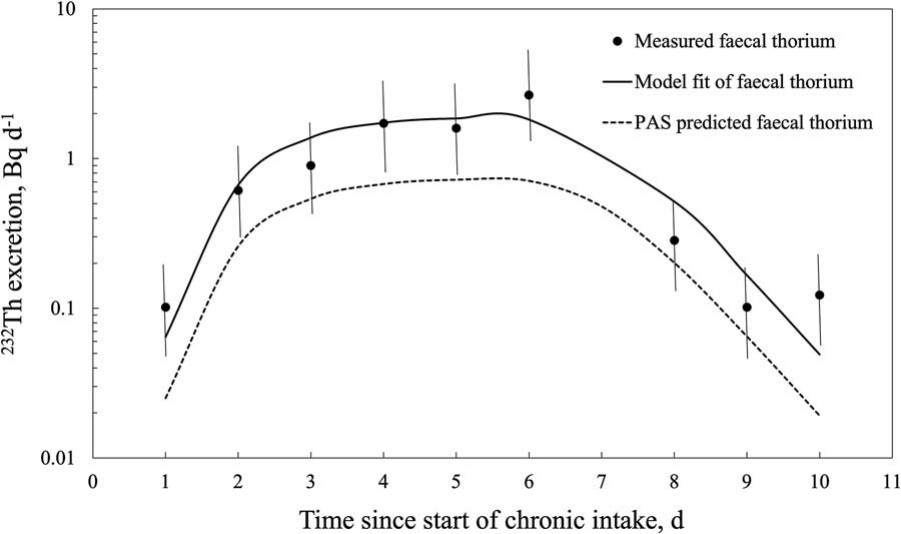
Retrospective fitting of faecal thorium bioassay data from worker E2 vs PAS-predicted faecal excretion. Uncertainty shown as a scattering factor of 2.

Figures 3 and 4 show the outputs from the retrospective bioassay fitting function of the Taurus software with the thorium faecal excretion data shown in Table 1 for workers E1 and E2, respectively. For comparison, the prospective bioassay curve based on PAS data (i.e. 1.25 Bq d^−1 232^Th intake) is also shown. Table 2 summarizes the predicted ^232^Th intake and committed effective dose derived from the faecal excretion data for both workers and the two modes of breathing (i.e. nose and mouth). The goodness-of-fit statistics for the Taurus-derived curves are also listed.

**Table 2:** Predicted intakes and effective doses derived from bioassay assessment.

^232^Th intake scenario^a^	Exposure parameter	Worker E1	Worker E2^b^
10 μm AMAD, type S, mouth breather, heavy work	^232^Th intake over exposure period (Bq d^−1^)	3.66	3.46
	Effective dose^c^ (mSv)	2.40	2.20
10 μm AMAD, type S, nose breather, heavy work	^232^Th intake over exposure period (Bq d^−1^)	3.23	3.21
	Effective dose^c^ (mSv)	0.96	0.95
Goodness of fit (chi-square) statistics	Total deviance (*χ*^2^)	1.35	4.16
	Probability	0.85	0.84

^a^Aerosol assumed to be monazite dust with shape factor of 1.25, particle density of 5.0, and size distribution with GSD of 3.0.

^b^Original measured faecal excretion for Days 7 and 8 combined due to very low faecal ash weight (4.1 g) of sample collected on Day 7.

^c^Effective dose over the 5-d exposure period for the ^232^Th series assuming secular equilibrium.

The Taurus model fit predictions for both workers were in very good agreement with the measured faecal excretion rates. Worker E1’s data provided a good fit, even though faecal samples were collected every second day over the 10-d monitoring period. If the Day 7 and 8 faecal collections for worker E2 had not been combined, the model predictions would not have aligned as well with the measured data, but there was good agreement for the first 6 d. The observed data after Day 7 may represent a residual thorium excretion rate following long-term chronic intake, together with dietary intake.

**Figure 5:**
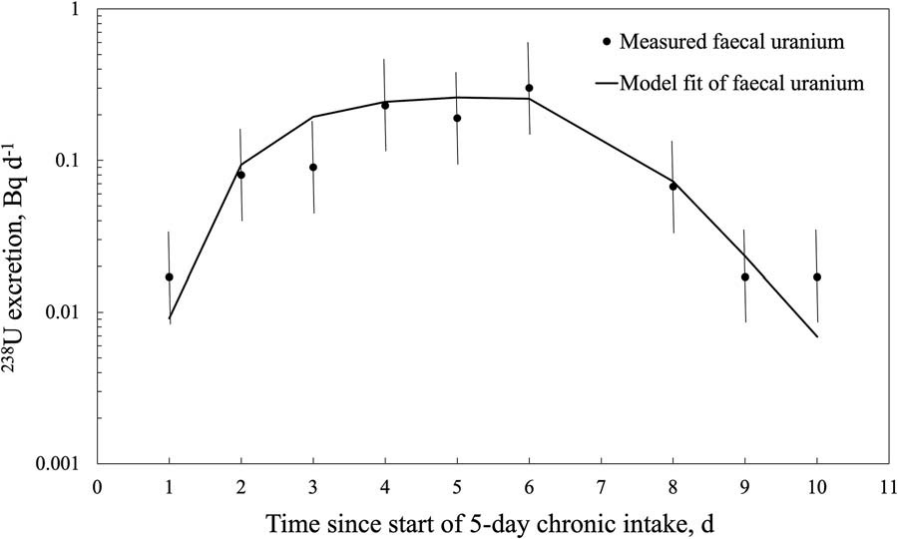
Retrospective fitting of faecal uranium bioassay data from worker E2 (20 mBq d^−1^ was subtracted for background correction). Uncertainty shown as a scattering factor of 2.

Figure 5 shows the Taurus fitting function analysis for worker E2’s measured ^238^U faecal excretion data. The model fit resulted in an estimated daily intake of 0.45 Bq d^−1^ over the 5-d exposure period (i.e. one-eighth of the ^232^Th intake). The resulting 5-d dose from the natural uranium series was 0.084 mSv for a nose-breathing worker doing heavy work and by applying the derived dose conversion factor (3.75E−02 mSv Bq^−1^) for type S, 10 μm AMAD monazite dust. Worker E2’s dose from the uranium series intake was 11-fold lower than the dose from the thorium series intake.

## Discussion

An interesting observation arising from a reanalysis of the original faecal thorium and uranium activities (Table 1) is the measured ratios of thorium to uranium. The activity ratios from Days 2 to 6 (i.e. 6:1 to 8:1) clearly indicated occupational exposure to monazite and other mineral sands dust, given that monazite has a Th:U activity ratio of ~10:1 [2]. Prior to and following the cessation of exposure at work, the ratios (~3:1 or less) reflect activity ratios in typical soils and rocks in Western Australia [12].

The prospective model predictions for chronic inhalation of thorium-bearing dust (Fig. 1) indicated only small differences in the expected peak daily thorium excretion, irrespective of particle size (AMAD) or mode of breathing. However, following cessation of intake, significant differences in excretion rates were noted after 5 d depending on the AMAD and mode of breathing. This finding highlights that samples collected following time off from work will likely provide significantly different estimates of intake and dose, depending on when the sample is collected and the assumptions around intake parameters. Figure 2 further reinforces this point and illustrates the impact of work–rest cycles on excretion rates.

Figure 2 shows that following chronic intake of relatively insoluble thorium over a 10 d on and 4 d off work cycle, the daily activity excretion rate quickly approached ~60% of the daily intake by Day 5 and peaked after the end of the exposure period (Day 10). Hence, a 24-h faecal sample collected between Days 5 and 11 of the work cycle is predicted to return the same estimate of daily thorium intake and, hence, the effective dose. A sample collected in the 24 h immediately after exposure ceases (i.e. Days 10 and 11) may be a useful collection strategy given that there is likely to be a lower risk of external contamination from the workplace, body, and work clothing. The faecal thorium excretion rate drops rapidly after workplace exposure ceases, and the length of the rest cycle determines the residual thorium excretion rate.

The bioassay-derived intakes and effective doses were very similar between the two workers (Table 2). In contrast, Terry *et al.* [10] applied the ICRP 30 internal dosimetry models to the same bioassay data set and determined that worker E2 had twice the intake of thorium of worker E1. The derived intakes in this study were similar for both the nose- and mouth-breathing scenarios; however, the dose was ~2.5 times higher if the worker was a mouth breather. This finding underlines the need to confirm the breathing mode when conducting individual monitoring. The estimated dose for the intake of insoluble NORM dust is also strongly dependent on the aerosol particle size distribution; hence, it is important to use site- and material-specific data when interpreting bioassay data.

The bioassay-derived effective dose of ~1 mSv for a nose-breathing worker over a 5-d (i.e. one working week) exposure period infers a significant annual dose to workers if the workplace airborne radioactivity concentrations remain relatively constant over the year. However, the study involved two volunteers working full-time in a monazite plant and without the use of respiratory protection. Hence, the study exposure scenario represented the worst case, and actual worker intakes were likely to be lower after accounting for task rotation and intermittent respirator use.

A substantial discrepancy was noted between the PAS-derived intake and dose when compared with the values derived from the bioassay assessment. The PAS intake obtained from daily sampling during the 5-d exposure period was 1.25 Bq d^−1^ of ^232^Th, which was 2.5–3 times lower than the intake obtained by applying the thorium biokinetic model to the faecal excretion data. Changing the aerosol AMAD between 5 and 20 μm did not materially affect the bioassay-predicted daily intake, and a possible reason for this discrepancy is that the PAS methodology used may not provide representative estimates of thorium intake. Previous bioassay studies of lung burdens of Western Australian mineral sands workers also found that PAS-derived estimates of intake underestimated doses [16].

The sampling protocol for measuring airborne radioactivity in Western Australian NORM industries has remained essentially unchanged for more than 30 y. A local regulatory guideline specifies the use of a seven-hole sampler [17], which was the sampler used in the faecal bioassay study. Past research on Western Australian mineral operations found that the seven-hole sampler underestimated the inhalable aerosol fraction by approximately two-fold, especially when large dust particles (>15 μm) were present [18]. This finding is consistent with the study by Kenny *et al.* [19], which showed a significant drop-off in the collection efficiency of the seven-hole sampler at particle aerodynamic diameters above ~20 μm. The workplace aerosol distribution in the faecal bioassay study was characterized by a relatively large AMAD (14 μm) [10]. Other particle size studies at Western Australian mineral sands operations using personal cascade impactors attached to workers consistently showed AMADs of 10 μm or greater [18, 20]. Further underestimation of the airborne radioactivity concentration may be due to the lack of accounting for sampler head wall losses, where a portion of the airborne dust adheres to the sampler walls and is not included in filter-based radioactivity measurements. The dust sample collected by PAS is analysed by alpha particle counting and alpha particle self-absorption may also be a contributing factor if dust loadings on the filter paper exceed 1 mg cm^−2^ [21]., Thus, the lower PAS-derived intake may be explained by a combination of air sampler bias, wall loss, and alpha self-absorption. PAS intake estimates may be improved by use of alternate dust samplers with collection characteristics that more closely conform to the inhalable aerosol convention specified by the International Organisation for Standardisation [22].

The contribution of thorium intake from environmental sources, including diet, or contamination during sample collection could be further factors resulting in the discrepancy observed between PAS- and bioassay-derived intake estimates. However, occupational thorium intake by a mineral sand worker is orders of magnitude above the worker’s likely environmental intake [14]. Contamination during sample collection is reduced by emphasizing the need for high standards of personal hygiene. The goodness-of-model fit to the measured data (Figs 3 and 4) and the absence of anomalously high measurements indicate that contamination was unlikely a factor in the study.

There are relatively few published studies [23, 24] on faecal thorium excretion by occupationally exposed workers and meaningful comparisons are complicated by the differences in the timing and duration of sample collection. Collection after a few days of chronic exposure should represent a peak excretion rate and provide a measure of the fraction of inhaled thorium-bearing dust that is cleared rapidly. Collection during time off work is strongly dependent on the time since the last exposure (Figs 1 and 2). The International Atomic Energy Agency (IAEA) recommends faecal collections over 3 to 4 d and, in the case of NORM-related exposures, suggests the collection should be after 10 d of absence from occupational exposure [25]. The IAEA rationale is that post-time-off-work measurements reflect the delayed clearance of systemic activity and avidly retained naturally occurring radionuclides in the lungs. However, the cyclic nature of work exposures and the variable predicted excretion rates after 5 d of cessation of exposure shown in Figs 1 and 2 seemingly question the validity of such a measurement strategy.

An important factor in the interpretation of bioassay measurements from NORM-exposed workers is the potential contribution of environmental sources, particularly diet. The EURADOS guidelines on individual monitoring list background uranium and thorium activity concentrations in faeces found in various studies [14]. Excluding measurements taken from residents living in high natural background areas, the mean daily faecal excretion of ^232^Th and ^238^U varied from 3.4 to 12 mBq and 13.5 to 28.5 mBq, respectively. As shown in Table 1, the excretion values before and after intake of ^232^Th (~100 mBq d^−1^) for workers E1 and E2 were well above the values reported for unexposed individuals. For ^238^U, both workers had before and after intake excretion values (37 mBq d^−1^) of a similar order to the reported background levels. Hence, in the case of the uranium bioassay values for monazite plant workers, the residual excretion rate arising from diet and the environment will affect the assessment of dose. In Fig. 5, a residual rate of 20 mBq d^−1^ of ^238^U was subtracted from the measured values to account for environmental exposure. This is a source of potentially significant uncertainty, and future bioassay studies should include a control group to confirm the background rates of excretion of thorium and uranium.

The geometric mean (GM) daily faecal excretion of ^232^Th during the 5-d exposure period in this study (170 Bq mg_ash_^−1^, GSD = 2.5) was higher than that reported in some other studies, which is likely due to the prevalence of coarse aerosols in the workplace, as evidenced by the measured AMAD. Juliao *et al.* [23] collected 24-h faecal samples from 20 monazite extraction plant workers in Brazil and reported ^232^Th values between 4.1 and 73 Bq mg_ash_^−1^ with a GM of 19 Bq mg_ash_^−1^ (GSD = 2.2). Lipsztein *et al.* [24] reported faecal ^232^Th concentrations of 10–240 mBq d^−1^ from a Brazilian niobium mine and found no correlation between excreta and PAS results. These activity concentrations were much lower than the peak daily concentrations found for the two Western Australian monazite plant workers (1550 and 2650 mBq d^−1^). The niobium mine study [24] reported the use of cyclones for PAS (i.e. collection of the respirable fraction of inhaled dust); hence, the PAS results will have underestimated exposure to coarse particles that are rapidly cleared via the faecal pathway. Lipsztein *et al.* [24] also measured faecal thorium excretion from the niobium mine workers after a vacation period of 30 d and reported that activity concentrations varied from the detection limit up to 11.7 mBq d^−1^ (i.e. consistent with background excretion rates).

In relation to feasibility of faecal thorium bioassay for the contemporary mineral sands industry workforce, Ralph *et al.* [26] report an average annual dose of ~1 mSv from airborne radioactive dust in mineral sands and rare earth processing operations in Western Australia. Maximum annual doses ranged from 2.7 to 4.2 mSv over the 3-y period 2020/21 to 2022/23. The average dose estimate implies an average chronic thorium intake of 0.05 Bq d^−1^ for a 10 μm AMAD, type S aerosol (or 0.025 Bq d^−1^ for 5 μm AMAD). Figure 2 shows that 60% of the daily thorium intake is excreted in faeces, which would correspond to 30 mBq d^−1^ and perhaps double this value for workers with higher exposure. Activity concentrations of this order can be measured; however, the influence of environmental exposure is likely to be significant at such concentrations. Hence, it will be important to understand the faecal thorium excretion rates of occupationally unexposed individuals living in similar regions to the workers. Ralph *et al.* [26] also highlighted that some Western Australian mineral sands operations have recently recommenced the processing of thorium-rich materials (30% monazite) to extract rare earth minerals. This has resulted in an increase in the maximum reported airborne radioactivity levels and may lead to increased doses over time, hence providing justification to revisit the feasibility of faecal bioassays. However, a significant consideration is worker aversion to participating in faecal sampling, and so such sampling may be more suited to abnormal exposure situations.

## Conclusions

The findings from this study indicate that thorium intakes and the corresponding radiation doses estimated through faecal bioassay were significantly higher than those derived from PAS, suggesting that PAS may not fully capture inhalation exposure in certain work environments (e.g. where coarse aerosols are prevalent). This study also identified critical factors influencing faecal excretion patterns, including work–rest cycles, mode of breathing, and dietary thorium intake.

Retrospective data fitting using the latest ICRP models demonstrated excellent agreement with measured faecal excretion data. Retrospective bioassay assessments were shown to be sensitive to model input parameters such as the mode of breathing, aerosol particle size, and aerosol solubility, and hence such parameters should be selected based on material-specific and individual data to the extent feasible.

Faecal bioassay has value as a complementary monitoring technique, particularly for assessing exposure to insoluble mineral dust containing naturally occurring radionuclides. However, the feasibility of applying faecal bioassays in contemporary NORM exposure situations depends on practical considerations, such as worker compliance, optimizing the timing of bioassay sample collection, and the ability to differentiate occupational exposure from background environmental sources.

Future research should focus on improving PAS methodologies, including the use of alternate dust sampling devices, and evaluating specific individual characteristics (e.g. mode of breathing) during individual monitoring campaigns. Retrospective assessment of past bioassay studies (e.g. urine bioassay, *in vivo* lung counting or thoron-in-breath measurements) using updated internal dosimetry software could be investigated to validate the faecal bioassay findings. While this study reviewed data from only two workers, the internal dosimetry methodology and interpretation of results have application to faecal bioassay studies of other NORM-exposed workers.

## Ethics

The analysis of past data reported in this paper is deemed an Exempt Application under the Edith Cowan University Research Ethics Management System (REMS). This paper summarizes the data from bioassay investigations of two Western Australian mine workers conducted more than 20 years ago. The data at that time were obtained with the informed written consent from the participating workers, and this consent was provided for grouped data with no worker or employer identification to be published and made available to other interested parties. This study also accessed data from similar industries using publicly available scientific papers and research reports.

## References

[1] Hewson GS, Ralph MI, Cattani M. Impact of changes to International Commission on Radiological Protection models on occupational thorium ore dust intake. Rad Prot Dosim 2025;201:420–31. 10.1093/rpd/ncaf031 PMC12012373 40173079

[2] Hewson GS, Ralph MI, Cattani M. Thorium ore dust research applicable to mineral sands industry workers. J Radiol Prot 2025;45:011502. 10.1088/1361-6498/adacf6 39842036

[3] International Commission on Radiological Protection . Occupational intakes of radionuclides: part 1. ICRP publication 130. Ann ICRP 2015;44:1–192. 10.1177/0146645315577539 26494836

[4] International Commission on Radiological Protection . Occupational intakes of radionuclides: part 3. ICRP publication 137. Ann ICRP 2017;46:1–486. 10.1177/0146645317741391 29380630

[5] International Commission on Radiological Protection . Occupational intakes of radionuclides: part 4. ICRP publication 141. Ann ICRP 2019;48:1–497. 10.1177/0146645319834139 31850780

[6] International Commission on Radiological Protection . Occupational intake of radionuclides—OIR electronic annex distribution set. ICRP Dose Viewer. Ottawa: ICRP, 2022.

[7] International Atomic Energy Agency . Radiation protection and NORM residue management in the production of rare earths from thorium containing minerals. In: Safety Report Series, No. 68. Vienna: IAEA, 2011.

[8] Stradling N, Hodgson A, Phipps A. et al. Can low doses from inhaled natural thorium be confirmed by personal monitoring? In: Proc. 9th Int. Conf. on Health Effects of Incorporated Radionuclides: Emphasis on Radium, Thorium, Uranium and their Daughter Products. GSF–National Research Center for Environment and Health: Neuherberg, 2005.

[9] International Commission on Radiological Protection . Human respiratory tract model for radiological protection. ICRP publication 66. Ann ICRP 1994;24:vii. 10.1016/0146-6453(94)90018-3 7726471

[10] Terry KW, Hewson GS, Meunier G. Thorium excretion in feces by mineral sands workers. Health Phys 1995;68:105–9. 10.1097/00004032-199501000-00014 7661919

[11] International Commission on Radiological Protection . Limits for intakes of radionuclides by workers. ICRP publication 30. Ann ICRP 1979;3:i–ii. 10.1016/0146-6453(79)90122-2 20863798

[12] Twiss P, Watling RJ, Delev D. Determination of thorium and uranium in faecal material from occupationally-exposed workers using ICP-MS. At Spectrosc 1994;15:36–9

[13] UK Health Security Agency. Taurus Internal Dosimetry Software. Chilton: UK Health Security Agency, 2024.

[14] Castellani CM, Marsh JW, Hurtgen C. et al. IDEAS guidelines (version 2) for the estimation of committed doses from incorporation monitoring data. EURADOS report 2013-01. European Radiation Dosimetry e.V., 2013. 10.1093/rpd/ncv457 26541189

[15] International Commission on Radiological Protection . Basic anatomical and physiological data for use in radiological protection: reference values. ICRP publication 89. Ann ICRP 2003;32:1–282. 8659813

[16] Hewson GS . Inhalation and retention of thorium dusts by mineral sands workers. Ann Occup Hyg 1997;41:92–8

[17] Government of Western Australia . Managing naturally occurring radioactive material (NORM) in mining and mineral processing—guideline. NORM 3.4 monitoring NORM—airborne radioactivity sampling. Government of Western Australia, 2010.

[18] Terry KW, Hewson GS, Rowe MB. Characterisation of inhaled dust on mine sites. Report no. 195. Minerals Research Institute of Western Australia, 1998.

[19] Kenny L, Aitken R, Chalmers C. et al. A collaborative European study of personal inhalable aerosol sampler performance. Ann Occup Hyg 1997;41:135–53. 10.1016/S0003-4878(96)00034-8 9155236

[20] Koperski J . Particle size analyses in and around mineral sands operations. Rad Prot Australia 1993;11:126–32.

[21] Terry KW . Alpha self-absorption in monazite dusts. Health Phys 1995;69:553–5. 10.1097/00004032-199510000-00016 7558848

[22] International Organisation for Standardization . ISO 7708: Air quality—particle size fraction definitions for health-related sampling. Geneva: ISO, 1995.

[23] Juliao LQC, Lipsztein JL, Azeredo AMG. et al. Thorium workers’ bioassay data. Radiat Prot Dosim 1994;53:285–8. 10.1093/rpd/53.1-4.285

[24] Lipsztein JL, Melo DR, Sousa W. et al. NORM workers: a challenge for internal dosimetry programmes. Radiat Prot Dosim 2003;105:317–20. 10.1093/oxfordjournals.rpd.a006247 14526977

[25] International Atomic Energy Agency . Occupational radiation protection. General safety guide no. 7. IAEA, 2018.

[26] Ralph MI, Koshy J, Foley P. A review of radiation doses and associated parameters in Western Australian mining operations (2020-23). J Radiol Prot 2024;44:032501. 10.1088/1361-6498/ad6f1a 39142297

